# A comparative evaluation of laparoscopic TAPP videos on YouTube and WebSurg: surgical steps and educational value

**DOI:** 10.1007/s10029-025-03341-8

**Published:** 2025-05-03

**Authors:** Alisina Bulut, Muhammed İkbal Akın

**Affiliations:** 1 Department of General Surgery, Marmara University Faculty of Medicine, Basıbuyuk Yolu No: 9 D:2, Maltepe, Istanbul, 34854 Turkey; 2 Department of Gastrointestinal Surgery, Kosuyolu High Specialization Education and Research Hospital, University of Health Sciences, Istanbul, Turkey

**Keywords:** Laparoscopic TAPP, WebSurg, YouTube, Hernia repair, Inguinal hernia

## Abstract

**Purpose:**

Laparoscopic Transabdominal Preperitoneal (TAPP) videos on YouTube and WebSurg have an important place in surgical training. However, there are differences between these platforms in terms of training quality and compliance with standard surgical steps. The aim of this study was to compare laparoscopic TAPP videos on YouTube (limited to individually uploaded content) and WebSurg in terms of surgical technique and educational quality.

**Methods:**

Twelve videos meeting specific criteria were selected from both platforms. The 2 groups were compared using the 9 Commandments, which assessed compliance with surgical steps, and the Procedure Presentation Score (PPS), which assessed video quality/educational content. Data on video characteristics, such as view count, publication date, and duration, were also collected.

**Results:**

Although YouTube videos reached more viewers, WebSurg videos had higher compliance with the 9 Commandments (WebSurg median score 8/9 vs. YouTube median score 5/9, *p* < 0.01). In addition, WebSurg videos had higher PPS scores (median: 8) than YouTube videos (median: 5) (*p* = 0.02).

**Conclusion:**

When utilizing online video platforms for surgical training, institutional training platforms such as WebSurg should be preferred. When the videos included here were compared with YouTube in the light of the defined criteria; it was seen that YouTube videos were not of sufficient quality.

## Introduction

Groin hernias are a common challenge for surgeons, frequently requiring surgical attention. Each year, approximately 20 million groin hernia repairs are carried out globally [1], with inguinal hernia repair being the most frequently performed abdominal wall hernia procedure [2]. While the tension-free open repair technique, first introduced by Lichtenstein in 1989, remains widely utilized, minimally invasive approaches have gained prominence among surgical techniques over the past three decades [3, 4]. The laparoscopic TAPP (Transabdominal Preperitoneal) approach is also one of these minimally invasive surgical techniques.

As in many other fields, online videos have become an important tool in surgical education. Particularly, videos of laparoscopic surgeries, where the quality observed by the surgeon is preserved, make it feasible to conduct laparoscopic surgery training via video presentations. The nature of laparoscopic surgery makes it well-suited for creating audio-visual educational content. Modern laparoscopic equipment often includes built-in video recording capabilities, allowing for the straightforward production of high-resolution digital imagery [5]. However, a significant number of publicly accessible videos lack reliability, as many are not created by credible sources and may showcase techniques that are not evidence-based [6]. Additionally, some videos may include inaccurate or misleading promotional content [7, 8]. YouTube is the most commonly used video platform for this purpose [9, 10]. Its accessibility, free availability, and lack of peer review are key factors contributing to its popularity [11]. However, the absence of peer review and the lack of scientific validation raise concerns about the reliability of the education provided through YouTube videos. In contrast, platforms like WebSurg, which are specifically aimed at surgeons and focus primarily on contributing to educational content, offer a more reliable alternative. These platforms are more credible, as they feature standardized videos that have undergone peer review and are designed to enhance surgical education [12, 13].

There is a limited number of studies in the literature that evaluate laparoscopic TAPP videos on YouTube based on specific criteria [14, 15]. However there is no existing study focused specifically on WebSurg. Noting this gap in the literature, we aimed to compare laparoscopic TAPP videos available on YouTube and WebSurg in terms of surgical techniques and visual clarity. In this study, only individually uploaded YouTube videos were evaluated, and institutional or organization-affiliated videos were not included in the analysis.

## Methods

### Video selection

On November 4, 2024, a search for the term ‘’ Trans-Abdominal Preperitoneal Hernia Repair ‘’ and “TAPP procedure” was first conducted on the WebSurg platform, as it was anticipated that fewer relevant videos would be available. Videos involving hernia repairs other than inguinal hernia (e.g., Spigelian hernia, lumbar hernia, umbilical hernia, ventral hernia, obturator hernia, femoral hernia), as well as recurrent hernia repairs, robotic surgeries, single-port surgeries, videos presented as slideshows, or those containing only a specific segment of the surgical procedure, were excluded. Following this selection process, 12 suitable videos were identified and added to a playlist, arranged in descending order based on their view counts. Subsequently, the keywords “Trans-Abdominal Preperitoneal Hernia Repair” and “TAPP procedure” were used to perform a search on YouTube. The search results were sorted by view count in descending order. Videos meeting the following criteria were selected: they showcased the entire laparoscopic TAPP procedure, were of assessable video quality, were not produced by commercial companies or educational institutions (considering they are assumed to have undergone quality review), were narrated in English, and did not include animations. The first 12 videos satisfying these criteria were compiled into a playlist. For each selected video, data regarding the publication date, view count, duration, and the number of likes and comments were recorded for subsequent analysis.

### Video evaluation

The videos were evaluated from two different perspectives. First, they were scored based on the adequacy of the surgical technique demonstrated and its adherence to established standard steps. For this scoring, the 9 Commandments, previously defined in the literature and developed by hernia surgery experts, were utilized as the evaluation framework [16]. Each video was evaluated based on the 9 criteria defined in the 9 Commandments. For each criterion, a score of 1 was assigned if the video met the standard, and 0 if it did not. Consequently, each video received a total score ranging from 0 to 9, reflecting its overall compliance with these criteria. Additionally, a review of the literature identified several key indicators of an effective educational surgical video. These included providing information about the preoperative preparation process, clearly identifying anatomical landmarks, explaining potential pitfalls, ensuring camera clarity, and presenting the procedure in a step-by-step manner. Although these criteria are not part of the 9 Commandments, they were deemed important for this study and were incorporated into a separate scoring system. We named this scoring system the Procedure Presentation Score (PPS). A second evaluation table was created for this purpose, including a total of five criteria. Each criterion was assigned a score ranging from 0 (lowest) to 2 (highest), resulting in a total possible score of 0–10 per video. All evaluations were conducted independently by two general surgeons who had participated in more than 100 hernia surgeries (including laparoscopic TAPP operations). The evaluators scored the videos separately, without knowledge of each other’s assessments, ensuring an unbiased evaluation process. In cases where the two evaluators assigned different scores, the median of the two values was calculated. If the median resulted in a decimal, it was rounded down to the nearest whole number. The median scores assigned by two independent observers were calculated for each of the 9 criteria included in the 9 Commandments and the 5 criteria of the PPS. As with the overall scores, fractional values were rounded down to the nearest whole number. The two video platforms were then compared individually for each of these 9 and 5 criteria.

### Statistical analysis

The statistical analysis was performed using Jamovi 2.3.28 software. As the data did not follow a normal distribution, the median and interquartile range (IQR) were calculated for the relevant variables. To compare the medians of independent groups, the Mann-Whitney U test was applied. For the scores, the median and IQR were also computed, and the Mann-Whitney U test was used to compare the medians between the two groups. For inter-observer reliability, Cronbach’s alpha was calculated, with values above 0.7 indicating acceptable agreement. A p-value of less than 0.05 was considered statistically significant.

Since this study did not involve interactions with patients or the use of patient health information, it was not subject to institutional review board approval.

## Results

A search conducted on WebSurg using the keyword “TAPP” identified 81 videos, while a search with the term “Trans-Abdominal Preperitoneal Hernia Repair” returned 27 videos. Some videos appeared in both search results, and certain links contained multiple video contents within a single title. Among the 81 videos categorized under the “TAPP” heading, 35 were found to involve total extraperitoneal (TEP) procedures (including eTEP and robotic approaches), 20 featured non-inguinal hernia surgeries, 7 were slideshow presentations, 4 demonstrated repairs for recurrent hernias, 2 utilized the single-port technique, and 1 depicted only a segment of the surgical procedure. Following this evaluation, 12 videos that were deemed appropriate were included in the study. For YouTube videos, the characteristics of excluded videos were not specifically detailed. Instead, the study focused exclusively on the 12 most-viewed videos that met the inclusion criteria. When comparing the two groups in terms of video age, view count, and video duration, a statistically significant difference was observed only in view counts, favoring YouTube videos. The median (IQR) view count was 167,259 (268,488) for YouTube videos, compared to 19,500 (30,075) for WebSurg videos (*p* < 0.001) (Table 1).

**Table 1:** Video characteristics

Parameter	WebSurg (median [IQR])	YouTube (median [IQR])	*p*-value
Video age (years)	11.3 (7.95)	6.95 (3.68)	0.14
View count (n)	19,500 (30,075)	167,259 (268,488)	< 0.001*
Length (minutes)	17.3 (10.8)	16.3 (14.4)	0.79
Likes (n)	N/R	1,300 (1,414)	-
Comments (n)	N/R	71.5 (121)	-

*N/R: Not Reported

When comparing the videos on two different platforms in terms of the 9 Commandments scores, WebSurg videos were found to be statistically significantly more compliant with the 9 Commandments. The median compliance score for WebSurg videos was 8/9, whereas for YouTube videos, it was 5/9 (*p* < 0.01). Both groups were also evaluated separately for each criterion within the 9 Commandments. No statistically significant differences were found between the two groups for 6 of the criteria. However, significant differences favoring WebSurg were observed for the criteria “Isolation of cord structures,” “Appropriate fixation,” and “Appropriate mesh placement,” with p-values of 0.03, < 0.01, and 0.01, respectively (Table 2).

**Table 2:** Comparison of “the 9 commandments” compliance scores between WebSurg and YouTube videos

Criterion	WebSurg (median[IQR])	YouTube (median [IQR])	*p*-value
Overall “The 9 Commandments” Score	8 (1)	5 (2)	< 0.01*
**The 9 Commandments**			
1.Wide medial dissection	1 (0)	1 (1)	0.07
2. Evaluate/reduce direct hernia	1 (0)	1 (1)	0.37
3. Space of Retzius dissection	1 (0)	1 (0)	0.16
4. Evaluate/reduce femoral hernia	1 (0)	0 (1)	0.11
5. Isolation of cord structures	1 (0)	1 (1)	0.03*
6. Evaluate/reduce cord lipoma	0 (1)	0 (0)	0.20
7. Space of Bogros dissection	1 (0)	1 (0.2)	0.30
8. Appropriate fixation	1 (0)	0 (1)	< 0.01*
9. Appropriate mesh placement	1 (0)	1 (1)	0.01*

The videos were also evaluated in terms of the Procedure Presentation Score (PPS). As a result of this scoring, WebSurg videos had a median score of 8 (IQR: 1), while YouTube videos had a median score of 5 (IQR: 4), with a statistically significant difference (*p* = 0.02), indicating that WebSurg videos received higher scores. When evaluated individually, WebSurg videos scored significantly higher in the “Pre-Surgery Briefing” and “Step-by-Step Approach” criteria. The median (IQR) score for “Pre-Surgery Briefing” was 2 (0) for WebSurg videos compared to 0 (1.25) for YouTube videos (*p* = 0.005). Similarly, the median (IQR) score for “Step-by-Step Approach” was 1 (1) for WebSurg videos and 1 (1) for YouTube videos, with a statistically significant difference (*p* = 0.044; due to differences in data distribution, including a lower minimum score, higher standard deviation, and a lower mean score in YouTube videos.) (Table 3).

**Table 3:** Comparison of procedure presentation score (PPS) between WebSurg and YouTube videos

Criterion	WebSurg (median[IQR])	YouTube (median [IQR])	*p*-value
Overall Procedure Presentation Score(PPS)	8(1)	5(4)	0.02*
**PPS Criteria**			
1. Pre-Surgery Briefing	2(0)	0(1.25)	0.005*
2. Landmarks	2(0.25)	1(1)	0.096
3. Pitfalls	2(1)	1(0)	0.124
4. Camera Quality	1(1)	1(1.25)	0.064
5. Step-by-Step Approachª	1(1)	1(1)	0.044*

ª Although the median and IQR values are identical for the “Step-by-Step Approach” criterion, statistical significance (*p* = 0.044) is due to differences in data distribution, including a lower minimum score, higher standard deviation, and a lower mean score in YouTube videos

Inter-rater reliability was also assessed since the videos were evaluated by two independent assessors. For the 9 Commandments, Cronbach’s alpha was calculated as 0.81, indicating good internal consistency. For the Procedure Presentation Score (PPS), Cronbach’s alpha was found to be 0.91, demonstrating excellent internal consistency.

## Discussion

Online platforms like YouTube and WebSurg play a significant role in learning the laparoscopic TAPP technique. The viewership data presented in this study highlights the considerable interest in these platforms, underscoring their role in filling a gap in surgical education, whether formally or informally. Adopting a conservative approach and dismissing the role of online educational materials would be inconsistent with the realities of modern times. The key lies in evaluating the conformity of such resources to standardized surgical steps, assessing their educational quality, and identifying deficiencies to contribute to their improvement. In the current study, although YouTube videos reached a significantly larger audience, we observed critical gaps in essential surgical steps. When analyzed against the 9 Commandments framework—a reference for steps to be followed during laparoscopic TAPP procedures—WebSurg videos demonstrated significantly higher compliance. A closer examination of the 9 Commandments revealed that the steps where WebSurg videos excelled over YouTube videos were particularly “Isolation of cord structures,” “Appropriate fixation,” and “Appropriate mesh placement.” These steps are indispensable for a safe and effective hernia repair. Inappropriate handling during cord isolation can lead to one of the most serious complications of hernia surgery: damage to the spermatic cord. Even with flawless dissections, improper mesh placement creates a significant risk for hernia recurrence. Similarly, inadequate mesh fixation not only increases recurrence risk but can also result in challenging complications such as bleeding, chronic pain, or paresthesia. These critical steps must be thoroughly understood by all young surgeons who are still progressing along their learning curve. Relying on videos with inadequate educational content in these areas may result in devastating outcomes for surgeons and their patients in the years to come.

There are various scales and scoring systems used in the literature to assess the adequacy of surgical videos. One of these is the Global Operative Assessment of Laparoscopic Skills-Groin Hernia (GOALS-GH). This tool is designed as an interactive, hands-on assessment method to evaluate the performance of trainees during laparoscopic inguinal hernia repairs. It not only gauges technical surgical skills but also assesses the surgeon’s comprehension of the procedure and their ability to maintain a consistent workflow. However, applying this tool to evaluate surgical videos poses significant challenges [17]. Huynh et al. [16]. reported that there is no universally validated scale for assessing minimal invasive hernia surgeries, with none being definitively the best. However, they acknowledged that the GOALS-GH comes closest to achieving this goal. Despite its merits, the tool’s evaluation focuses on only three major surgical steps—creation of workspace, reduction of the hernia sac, and mesh placement—rather than offering a detailed, step-by-step assessment. This limitation makes it less comprehensive for evaluating complex procedures. In their own study on evaluating minimal invasive hernia repair videos, they utilized the 9 Commandments. They emphasized that adherence to these steps reduces complications and recurrence risk, thus ensuring optimal patient care. The results from their study align with our findings, reinforcing the notion that while platforms like YouTube may be free and easily accessible, they may not always be reliable for educational purposes without a peer-review process [16]. This result is not surprising considering that the WebSurg platform was established by competent and authorized surgeons to improve surgical training processes. Furthermore, the acceptance of videos that meet specific quality standards after peer review increases the potential for these videos to align with standardized surgical teachings. In other studies in the literature comparing YouTube surgical videos to those on WebSurg, WebSurg has been reported to outperform YouTube in various aspects [18, 19]. In another study evaluating laparoscopic TAPP videos on YouTube, it was reported that these videos did not comply with the the LAParoscopic surgery Video Educational GuidelineS (LAP-VEGaS) and International Endohernia guidelines. The quality and utility of these videos as surgical learning tools were questioned [14].

One of the main reasons why YouTube videos are generally considered inadequate or unreliable is the lack of peer-review during the video upload process. Moreover, not every user uploading videos may prioritize providing high-quality educational material. Some may simply share videos of surgeries they have performed on their personal pages for self-satisfaction, or use them as a promotional tool, targeting a broader audience, which may not necessarily consist of surgeons but rather the general public. These factors may prevent the creation of meticulous, standardized, and safe surgical video presentations. Despite all these challenges, we cannot claim that all YouTube surgical videos are entirely inadequate or unnecessary. There are studies, albeit limited, that demonstrate some YouTube videos can be comparable to WebSurg in terms of their contribution to surgical education, showing that they are not necessarily inferior [20, 21]. In the study by Ferhatoglu et al. [21], which compared Sleeve Gastrectomy surgeries on YouTube and WebSurg using several scales, it was found that well-selected YouTube videos could be as good as WebSurg videos. However, there is an important point to highlight here. In this study, the highest-rated selected YouTube videos were compared with WebSurg videos that were not subject to any selection process. While it may be possible for experienced and established surgeons to select the best videos in terms of surgical quality and competence from among dozens or hundreds of YouTube videos, this selection may not always be as accurate for young surgeons who are still at the early stages of their careers and trying to learn surgery by watching these videos.

In laparoscopic TAPP repair, the 9 Commandments provide standardized steps to ensure a safe and effective surgery, and they are highly adequate in this regard. However, other scoring systems have also been used in the literature. Yağız et al. defined a 16-item video scoring system based on guidelines and literature [15]. In this system, factors such as patient positioning, surgeon and assistant positions, and the placement of a catheter in the bladder were scored separately. Additionally, in the LAP-VEGaS scoring system, criteria such as “The surgical procedure is presented in a standardized step-by-step fashion” and “The intraoperative findings are clearly demonstrated, with constant reference to the anatomy” are also evaluated [22]. In the present study, we sought to compare five extra criteria, not included in the 9 Commandments but deemed important, using the available literature. These criteria were preoperative preparation, demonstration of landmarks, explanation of pitfalls, video quality, and step-by-step presentation of the surgery. We aimed to make the comparison numerically by scoring these five parameters. Instead of evaluating these criteria as either sufficient or insufficient in a clear-cut manner, we opted for a scoring system from 0 to 2 to avoid missing out on nuances and to incorporate gray areas. To give this collective evaluation a name, we referred to it as the Procedure Presentation Score (PPS). When assessed in terms of PPS, we found that WebSurg videos scored significantly higher. In the comparison of individual criteria, YouTube videos received significantly lower scores, particularly in the “Pre-Surgery Briefing” and “Step-by-step Approach” categories. For these videos to effectively contribute to surgeons’ education in performing laparoscopic TAPP surgeries, it is crucial that they address key aspects such as trocar insertion points, the positioning of the surgeon, and the patient’s positioning during the initial steps. Moreover, to facilitate the learning process, presenting the surgical steps in a clear, step-by-step sequence in the video would enhance the comprehensibility of the procedure. In the study by Aktoz et al. [23], which compared laparoscopic hysterectomy in terms of educational reliability and quality, the LAP-VEGaS tool was used. WebSurg was found to be significantly superior in criteria such as “Position of patient, access ports, extraction site and surgical team,” “The surgical procedure is presented in a standardized step-by-step fashion,” and “The intraoperative findings are clearly demonstrated, with constant reference to the anatomy.” These findings align closely with the results of the PPS score for criteria 1 and 5 in the present study. However, we cannot conclude that the surgeons in YouTube videos do not appropriately or properly perform these steps. They may very well have applied these steps correctly and in an appropriate manner. The focus here is not on the competency of the surgeons performing the procedures but rather on the educational role of the videos.

To our knowledge, there are no studies in the literature evaluating laparoscopic TAPP videos and comparing them with YouTube versions. This is the first study comparing laparoscopic TAPP videos on WebSurg and YouTube. It is also one of the pioneering studies using the 9 Commandments as an evaluation tool. The fact that it compares the videos of the two platforms individually in terms of both the 9 criteria in the 9 Commandments and 5 other criteria defined in the literature are other features that add strength to the study. The evaluations were made by 2 competent surgeons blinded to each other’s scores is important to prevent possible biases. However, there are some limitations to this study. Although we believe that the sample size is sufficient when compared to similar studies, a larger number of videos would enhance the reliability of the results. The inclusion of only English-language videos is another limitation, as it may have excluded high-quality videos in other languages. Additionally, video acceptability for learning is a subjective process, influenced by factors such as the audience’s experience and the availability of similar content, which are not captured by the assessment tool. Moreover, the tool primarily evaluates technical skills and does not consider essential elements like teamwork, communication, and problem-solving in unexpected situations. It should also be noted that only individually uploaded YouTube videos were analyzed in this study, and institutional or organization-affiliated videos were not included. This may have led to a selection bias toward lower-quality content and represents a major limitation in terms of the generalizability of the results. Variations in TAPP techniques, such as self-fixating meshes, glue fixation methods, and the management of direct and inguinoscrotal sacs, were not addressed in the “commandments.” Due to these variables that we cannot include in the evaluation, it would not be correct to make large generalizations based on the data we have presented. In addition, the lack of other scores such as LAP-VEGaS and GOALS-GH, which are defined as video evaluation tools in the literature, are among the limitations.

## Conclusion

Surgical videos on platforms such as YouTube and WebSurg can easily reach thousands of viewers. For this reason, it should be recognized that they are important tools in surgical education. However, YouTube videos, which lack peer review and standardized selection criteria, may vary in reliability compared to videos from institutional platforms like WebSurg. Platforms like WebSurg primarily focus on surgical education and are designed to align with established guidelines, potentially offering a more structured and standardized resource for surgical training.

## Data Availability

All data generated or analyzed during this study are included in this published article.

## Declarations

### Permission to reproduce material

This manuscript does not include any material reproduced or adapted from other sources.
